# Karyopherins: potential biological elements involved in the delayed graft function in renal transplant recipients

**DOI:** 10.1186/1755-8794-7-14

**Published:** 2014-03-14

**Authors:** Gianluigi Zaza, Federica Rascio, Paola Pontrelli, Simona Granata, Patrizia Stifanelli, Matteo Accetturo, Nicola Ancona, Loreto Gesualdo, Antonio Lupo, Giuseppe Grandaliano

**Affiliations:** 1 Renal Unit, Department of Medicine, University-Hospital of Verona, Piazzale A. Stefani 1, 37126 Verona (VR), Italy; 2 Renal, Dialysis and Transplant Unit-Department of Emergency and Transplantation, University of Bari, Bari, Italy; 3 Istituto studi sui sistemi intelligenti per l′automazione (ISSIA), Consiglio Nazionale delle Ricerche (CNR), Bari, Italy; 4 Nephrology, Dialysis and Transplantation Unit, Dept. of Medical and Surgical Sciences, University of Foggia, Foggia, Italy

**Keywords:** Delayed graft function, Renal transplantation, Microarray

## Abstract

**Background:**

Immediately after renal transplantation, patients experience rapid and significant improvement of their clinical conditions and undergo considerable systemic and cellular modifications. However, some patients present a slow recovery of the renal function commonly defined as delayed graft function (DGF). Although clinically well characterized, the molecular mechanisms underlying this condition are not totally defined, thus, we are currently missing specific clinical markers to predict and to make early diagnosis of this event.

**Methods:**

We investigated, using a pathway analysis approach, the transcriptomic profile of peripheral blood mononuclear cells (PBMC) from renal transplant recipients with DGF and with early graft function (EGF), before (T0) and 24 hours (T24) after transplantation.

**Results:**

Bioinformatics/statistical analysis showed that 15 pathways (8 up-regulated and 7 down-regulated) and 11 pathways (5 up-regulated and 6 down-regulated) were able to identify DGF patients at T0 and T24, respectively. Interestingly, the most up-regulated pathway at both time points was *NLS-bearing substrate import into nucleus*, which includes genes encoding for several subtypes of karyopherins, a group of proteins involved in nucleocytoplasmic transport. Signal transducers and activators of transcription (STAT) utilize karyopherins-alpha (KPNA) for their passage from cytoplasm into the nucleus. *In vitro* functional analysis demonstrated that in PBMCs of DGF patients, there was a significant KPNA-mediated nuclear translocation of the phosphorylated form of STAT3 (pSTAT3) after short-time stimulation (2 and 5 minutes) with interleukin-6.

**Conclusions:**

Our study suggests the involvement, immediately before transplantation, of karyopherin-mediated nuclear transport in the onset and development of DGF. Additionally, it reveals that karyopherins could be good candidates as potential DGF predictive clinical biomarkers and targets for pharmacological interventions in renal transplantation. However, because of the low number of patients analyzed and some methodological limitations, additional studies are needed to validate and to better address these points.

## Background

Renal transplantation is the main treatment for advanced chronic kidney disease and it is associated with an improvement in the quality of life and survival of these patients compared to dialysis treatment [1-4]. Several evidences underline that immediately after transplantation, patients experience rapid and significant changes of their clinical conditions and undergo considerable physiological modifications [5,6]. These changes may be primarily induced by the kidney physiology reactivation, the ending of bioincompatible dialysis stimuli (e.g. microinflammation, oxidative stress) and the pharmacological effects following induction therapy [7,8].

Although several therapeutic strategies have been introduced to reduce early transplant complications [9-11], a significant number of patients experience a slow recovery of the renal function and they need to continue dialysis treatment after transplantation. This condition is commonly described as delayed graft function (DGF) [12]. DGF is a multi-factorial event influenced by several factors (e.g., kidneys from non-heart-beating donor, inotropic support of the donor), donor characteristics (e.g., age, diabetes, hypertension), graft functional features as shown by the transcriptomic profile of pre-transplant biopsy, recipients conditions (e.g. pre-transplant dialysis treatment, number of previous transplants, allosensitisation) and length of cold ischemia time [13].

Prolonged hypothermic ischemia and subsequent reperfusion may activate a complex sequence of events promoting renal damage and DGF. Ischemia starves tissue of oxygen and nutrients and causes accumulation of metabolic waste products [10], inhibition of oxidative metabolism, ATP depletion and increase in anaerobic glycolysis [11]. Then, in response to renal ischemia, cytoprotective machinery is activated including rapid decrease of cellular metabolic activity. Additionally, it is largely reported a significant cytoprotective or regenerative genes transcription [12]. Reinstitution of blood flow in ischemic kidneys causes a cascade of events, first and foremost the release of reactive oxygen species [12-14]. In addition, ischemia-reperfusion injury (IRI) is characterized by the immune system activation, including the local recruitment of monocyte/macrophages, granulocytes and dendritic cells [15-17]. Moreover, recently our group has demonstrated that DGF is associated with an increased T-bet/GATA-3 ratio in graft infiltrating CD4+ T cells, suggesting the priming of a Th1 response [18]. All these events create a biological background that makes the allograft more susceptible to develop both acute rejection and chronic allograft nephropathy with a consequent reduction of the graft survival [19,20].

Huge efforts have been made in the field of transplantation analyzing the organ-related, donors and recipients biological characteristics influencing DGF and to identify novel biomarkers that enable transplant clinicians the early identification of patients at high risk to develop this important complication [9].

The present study, utilizing non parametric permutation tests and statistically well founded approaches for the integrative analysis of genes and pathways assayed with high-throughput microarray technology, has been able to shed light on the molecular basis of DGF and to identify a specific transcriptomic signature potentially useful as predictive and early diagnostic biomarker in renal transplant recipients undergoing this important clinical complication.

## Methods

### Patients

In our study, we randomly selected 12 consecutive adult first renal transplant recipients who developed DGF and 12 with early graft function (EGF). DGF was considered as the need for dialysis within the first week of transplantation.

After enrollment, the 24 patients have been randomly split into two groups (Table 1):

1. *Training-group* that included 7 patients with DGF and 7 patients with EGF;

2. *Testing-group* that comprised 5 DGF and 5 EGF patients.

**Table 1:** Demographics and clinical characteristics of the two study groups

**Demographic/clinical characteristics**	**Training group**		**p value**	**Testing group**		**p value**
	**EGF**	**DGF**		**EGF**	**DGF**	
Patients (n)	7	7		5	5	
Gender (M/F)	4/3	3/4	n.s.	2/3	3/2	n.s.
Recipient age (years)	47.1 ± 9.3	48.3 ± 8.4	n.s.	46.3 ± 10.1	47.7 ± 7.5	n.s.
Donor age (years)	47.3 ± 9.3	51.9 ± 12.6	n.s.	49.1 ± 6.4	49.5 ± 5.9	n.s.
Donor cause of death (trauma/others)	3/4	3/4	n.s.	2/3	2/3	n.s.
HLA mismatches (n)	3.4 ± 1.2	3.5 ± 0.8	n.s.	3.4 ± 1.1	3.5 ± 0.9	n.s.
Cold ischemia time (h)	12.1 ± 3.8	17.4 ± 5.1	<0.05	11.2 ± 2.3	16.8 ± 4.3	0.01
Length of DGF (days)	0	17.8 ± 12.8	<0.001	0	14.2 ± 16.3	<0.001

EGF, early graft function; DGF, delayed graft function; HLA, human leukocyte antigen. Values are expressed as mean±SD. P-value calculated by T-test and Chi-square test.

DGF patients underwent a percutaneous graft biopsy to exclude acute rejection. All patients presented low panel reactive antibody (PRA) levels measured by Luminex.

Recipients of expanded criteria donors or having a very high pre-transplant cold ischemia time were excluded. Additionally, no patients included were affected by diabetes, chronic lung diseases, neoplasms, or inflammatory diseases.

To avoid drug-related confounding factors all enrolled patients received 500 mg of steroids IV and a single infusion of anti-CD25 at the time of transplantation.

Biological samples were collected just before transplantation (T0) and 24 hours after transplantation (T24).

The study was approved by the institutional ethical board of the University Hospital “Policlinico di Bari”, Bari, Italy and all patients signed an informed consent according to the last version of the declaration of Helsinki.

### Peripheral blood mononuclear cells (PBMC) isolation

Twenty ml of whole blood were harvested from all patients at the time of enrolment (T0) and 24 hours after transplantation (T24). Peripheral blood mononuclear cells (PBMC) were isolated by density separation over a Ficoll-PaqueTM (GE healthcare, Uppsala, Sweden) gradient (460 g for 30 min) and were washed three times with phosphate-buffered saline (PBS) pH 7.4/1 mM EDTA (Sigma, Milan, Italy). Cells were counted and their viability was evaluated by trypan blue exclusion (>90% PBMC were viable).

### RNA extraction and gene expression profiling

For all patients included in the training-group, total RNA was extracted from 10^6^ PBMCs at T0 and T24 using the RNeasy Mini Kit (Qiagen AG, Basel, Switzerland). The concentration of total RNA was measured using NANODROP® spectrophotometer ND-1000 (Thermo Scientific, Waltham, MA).

From all the 14 patients included in the training-group, RNA was processed and hybridized to a set of 14 arrays for the “T0 analysis” and other 14 arrays for “T24 analysis”. For our experiments we used the GeneChip® Human Genome U133A oligonucleotide microarray (Affymetrix, Santa Clara, CA) which contains 22,283 gene probe sets, representing 12,357 human genes, plus approximately 3,800 expressed sequence tag clones (ESTs), according to manufacturer’s instructions. We used the default settings of Affymetrix Microarray Suite software version 5 (MAS 5.0; Affymetrix) to calculate scaled gene expression values.

### Confocal microscopy

In order to analyze the activation of karyopherin-related machinery in DGF, we stimulated PBMCs with IL-6, a major activator of signal transducer. In particular, by binding its receptor IL6ST (GP130) and activating Janus kinases (JAK), it induces the phosphorylation and activation of STATs (signal transducer and activator of transcription), including STAT3 which then translocate to the nucleus and act on target gene transcription. This translocation is mediated by karyopherins-alpha.

Co-localization of KPNA (SRP) and either phospho-STAT3 or total STAT3 was evaluated by indirect immunofluorescence and confocal microscopy on PBMC isolated from 5 DGF and 5 EGF (testing-group), incubated with or without 50 ng/mL rh-IL-6 (Strathmann Biotec AG, Hamburg, Germany) for 2 min and 5 min, and spotted on poly-L-lysine-coated slides. We chose these two time points and this concentration after time-course and dose–response experiments (data not shown).

After fixing in 3.7% paraformaldehyde, cells were permeabilized in PBS with 0.25% TritonX-100 for 7 min, washed in PBS and then blocked with 2% bovine serum albumin (BSA) in PBS for 1 hour at room temperature. After blocking, the slides were incubated overnight with anti-phospho-STAT3 (1:100, mouse anti–human monoclonal sc-8059, Santa Cruz Biotechnology, Santa Cruz, CA) or anti-STAT3 antibody (1:100, rabbit anti-human polyclonal sc-482, Santa Cruz Biotechnology). The slides were then extensively washed in PBS and incubated with Alexa Fluor goat anti-mouse 488 (1:200, Molecular Probes, Eugene, OR) for 1 hour for double staining SRP/phospho-STAT3 and with Alexa Fluor goat anti-rabbit 488 (1:200, Molecular Probes) for double staining SRP/STAT3. After washings, the slides were blocked with BSA 2% for 1 hour and then incubated with anti-SRP antibody (1:500, mouse polyclonal anti-human ab55387, Abcam, Cambridge, UK). Sections were washed and then incubated with Alexa Fluor goat anti-mouse 555 (1:200, Molecular Probes). The nuclei were counterstained with To-pro-3 (Molecular Probes). The slides were finally mounted with GEL/MOUNT (Biomeda Corp., Foster City, CA). Negative controls were performed by omitting the primary antibodies. Fluorescence was acquired by a Leica TCS SP2 (Leica, Wetzlar, Germany) confocal laser-scanning microscope.

### Statistical analysis

Data are expressed as the mean ± standard deviation (SD). T-test and chi-square test were used to assess differences in clinical and demographic features. A value of p < 0.05 was considered to be statistically significant.

For microarray analysis, gene expression values for the 22,283 gene probe sets, scaled to the target intensity of 2,500, were log transformed. To identify significant transcriptomic differences between DGF and EGF patients (at both T0 and T24) and to overcome many of the drawbacks associated with standard approaches based on single gene analysis [21], we used Gene Set Enrichment Analysis (GSEA) [22], a statistically well founded method which finds pathways and biological processes enriched of differentially expressed genes in two different phenotypic conditions. GSEA is a computational method that determines in silico whether a group of genes included in the same biological process (pathway) shows statistically significant differences between two biological conditions (phenotypes). This method uses a variation of a Kolmogorov-Smirnov statistic to provide an enrichment score for each gene set. We use the signal-to-noise metric in the standard GSEA setting as our score. These enrichment scores are then normalized to take into account the size of the gene sets resulting in a normalized enrichment score. We performed 1000 random permutations of the phenotypic labels to compute p-value and false discovery rate (FDR).

For our analysis, we used the global expression level of 825 biological processes listed in the c5 collection of Molecular Signature Database (MSigDB) (http://www.broadinstitute.org/gsea/msigdb/index.jsp) and the algorithm of analysis described by Subramanian et al. [22]. R 2.0.1 statistical software was used to perform the above analyses. Principal component analysis (PCA) was performed using Spotfire Decision Site 9.0 (http://www.spotfire.com).

## Results

### Demographic and clinical characteristics

As shown in Table 1, we did not find any significant difference between DGF and EGF patients in several demographic and clinical parameters (donor and recipient age, gender, number of HLA mismatch) in both training- and testing-group. Only the cold-ischemia time resulted significantly higher in DGF compared to EGF (p < 0.05) in both study groups. The length of DGF was 17.8 ± 12.8 and 14.2 ± 16.3 days (mean ± SD) in training- and testing-group, respectively.

### Identification of a specific pre-transplant transcriptomic fingerprint associated with DGF

In order to identify a pre-transplant (T0) PBMCs’ transcriptomic profiling associated with DGF, we compared the expression level of 825 pathways in DGF versus EGF patients.

After statistical analysis/bioinformatics, 15 pathways resulted differentially expressed in DGF versus EGF patients at T0 (p < 0.001, False Discovery Rate < 10%) (Table 2).

**Table 2:** Pathways discriminating patients developing delayed graft function (DGF) from those having early graft function (EGF) at the time of transplantation (T0)

**Pathway**	**Number of genes**	**Gene symbol**	**p value**
UP-REGULATED IN DGF			
NLS bearing substrate import into nucleus	13	CBLB, FYB, KPNA1, KPNA2, KPNA3, KPNA4, KPNA5, KPNA6, KPNB1, NCKIPSD, RANBP5, RERE, TRPS1	<0.001
Nuclear transport	89	AKT1, ALS2CR2, ANP32A, ATXN1, BARD1, BAT1, BCL3, BCL6, CALR, CBLB, CDH1, DDX19B, DDX25, DDX39, DUSP16, EIF5A, F2, F2R, FAF1, FLNA, FYB, GLI3, GSK3B, HNRNPA1, HRB, HTATIP2, KHDRBS1, KPNA1, KPNA2, KPNA3, KPNA4, KPNA5, KPNA6, KPNB1, LYK5, MALT1, MCM3AP, MDFI, MXI1, MYBBP1A, NCBP2, NCKIPSD, NF1, NFKBIE, NFKBIL1, NFKBIL2, NLRP12, NLRP3, NOP5/NOP58, NPM1, NUDT4, NUP107, NUP133, NUP160, NUP205, NUP214, NUP98, NUPL2, NXF5PDIA3, PPIH, PPP1R10, PTTG1IP, RAE1, RANBP2, RANBP5, RERE, RPAIN, SMAD3, SMG1, SMG5, SMG6, SMG7, TBRG1, TGFB1, TNF, TNFSF14, TNPO1, TPR, TRIP6, TRPS1, TSC1, UHMK1, UPF1, UPF2, XPO6, XPO7, ZFYVE9	0.004
Nucleocytoplasmic transport	88	AKT1, ALS2CR2, ANP32A, ATXN1, BARD1, BAT1, BCL3, BCL6, CALR, CBLB, CDH1, DDX19B, DDX25, DDX39, DUSP16, EIF5A, F2, F2R, FAF1, FLNA, FYB, GLI3, GSK3B, HNRNPA1, HRB, HTATIP2, KHDRBS1, KPNA1, KPNA2, KPNA3, KPNA4, KPNA5, KPNA6, KPNB1, LYK5, MALT1, MCM3AP, MDFI, MXI1, MYBBP1A, NCBP2, NCKIPSD, NF1, NFKBIE, NFKBIL1, NFKBIL2, NLRP12, NLRP3, NOP5/NOP58, NPM1, NUDT4, NUP107, NUP133, NUP160, NUP205, NUP214, NUP98, NUPL2, NXF5PDIA3, PPIH, PPP1R10, PTTG1IP, RAE1, RANBP2, RANBP5, RERE, RPAIN, SMAD3, SMG1, SMG5, SMG6, SMG7, TGFB1, TNF, TNFSF14, TNPO1, TPR, TRIP6, TRPS1, TSC1, UHMK1, UPF1, UPF2, XPO6, XPO7, ZFYVE9	0.004
Protein import into nucleus	48	AKT1, BCL3, BCL6, CBLB, CDH1, CEP57, F2, F2R, FAF1, FLNA, FYB, GLI3, KPNA1, KPNA2, KPNA3, KPNA4, KPNA5, KPNA6, KPNB1, MCM3AP, MDFI, MXI1, NCKIPSD, NF1, NFKBIE, NFKBIL1, NFKBIL2, NLRP12, NLRP3, NOP5/NOP58, NUP205, PDIA3, PPIH, PPP1R10, PTTG1IP, RANBP2, RANBP5, RERE, RPAIN, SMAD3, TGFB1, TNF, TNFSF14, TNPO1, TPR, TRIP6, TRPS1, ZFYVE9	0.004
Ribonucleotide metabolic process	16	ACLY, ADK, ADSS, AK5, AMPD3, C16orf7, CMPK, CTNS, CTPS, ENTPD4, FIGNL1, GUK1, NDUFS1, NUDT5, OLA1, UMPS	0.004
Nuclear import	50	AKT1, BCL3, BCL6, CBLB, CDH1, CEP57, F2, F2R, FAF1, FLNA, FYB, GLI3, HNRNPA1, HTATIP2, KPNA1, KPNA2, KPNA3, KPNA4, KPNA5, KPNA6, KPNB1, MCM3AP, MDFI, MXI1, NCKIPSD, NF1, NFKBIE, NFKBIL1, NFKBIL2, NLRP12, NLRP3, NOP5/NOP58, NUP205, PDIA3, PPIH, PPP1R10, PTTG1IP, RANBP2, RANBP5, RERE, RPAIN, SMAD3, TGFB1, TNF, TNFSF14, TNPO1, TPR, TRIP6, TRPS1, ZFYVE9	0.006
Pyrimidine nucleotide metabolic process	10	AK5, CMPK, CTPS, DCK, DCTD, ENTPD4, NT5C, NT5M, TYMP, UMPS	0.006
Macromolecule localization	237	ABCA1, ABCG1, ACHE, AGXT, AIP, AKAP10, AKT1, ALS2CR2, ANG, ANGPTL3, AP1G1, AP1GBP1, AP1M2, AP3B1, AP3D1, AP3M1, AP3S2, APBA1, APOA1, APOA2, APPBP2, ARCN1, ARFGAP3, ARFIP1, ARL4D, ATG4A, ATG4B, ATG4C, ATG4D, BACE2, BARD1, BAT1, BCL3, BCL6, BIN3, BIRC5, C3orf31, CADM1, CALR, CANX, CARD8, CBLB, CBY1, CD24, CD3G, CD74, CD81, CDC37, CDH1, CEP290, CEP57, CIDEA, CKAP5, COG2, COG3, COG7, COLQ, COX18, CRTAM, CTSA, CUTA, DDX19B, DDX25, DDX39, DERL1, DERL2, DNAJC1, DPH3, DUSP16, EGFR, EIF5A, ERCC3, ERP29, F2, F2R, FAF1, FLNA, FOXP3, FYB, GABARAP, GGA1, GGN, GLI3, GLMN, GSK3B, HNRNPA2B1, HOMER3, HPS4, HRB, ICMT, INS, KDELR1, KDELR2, KHDRBS1, KIF13B, KLHL2, KPNA1, KPNA2, KPNA3, KPNA4, KPNA5, KPNA6, KPNB1, LGTN, LMAN2L, LRP1B, LTBP2, LYK5, MAL, MCM3AP, MDFI, MFN2, MIPEP, MXI1, MYH9, MYO6, NAGPA, NCBP2, NCKIPSD, NF1, NFKBIE, NFKBIL1, NFKBIL2, NLGN1, NLRC4, NLRP12, NLRP2, NLRP3, NOD2, NOP5/NOP58, NPM1, NUDT4, NUP107, NUP133, NUP160, NUP205, NUP214, NUPL2, NXF5, OPTN, PDIA2, PDIA3, PDIA4, PEX1, PEX10, PEX12, PEX13, PEX14, PEX16, PEX19, PEX26, PEX3, PEX6, PEX7, PPIH, PPP1R10, PPT1, PPY, PTTG1IP, PYCARD, PYDC1, RAB35, RAB3GAP2, RAE1, RANBP2, RANBP5, REEP1, RERE, RPAIN, RPGR, RPL11, RTP1, RTP2, RTP3, RTP4, SCG2, SCG5, SEC23IP, SEC63, SELS, SERGEF, SHROOM2, SHR	0.008
DOWN-REGULATED IN DGF			
Vasculature development	55	ACVRL1, AGGF1, AMOT, ANG, ANGPTL3, ANGPTL4, ATPIF1, BTG1, C1GALT1, CANX, CCM2, CDH13, CHRNA7, COL4A2, COL4A3, CUL7, EGF, EGFL7, EMCN, EPGN, ERAP1, FOXC2, FOXO4, GLMN, HTATIP2, IL17F, IL18, IL8, MYH9, NCL, NF1, NOTCH4, NPPB, NPR1, PDPN, PF4, PLG, PML, PROK2, RASA1, RHOB, RNH1, ROBO4, RUNX1, SCG2, SERPINF1, SHH, SPHK1, SPINK5, STAB1, TGFB2, THY1, TNFSF12, TNNI3, VEGFA	0.005
Activation of protein kinase activity	28	ALS2CR2, ANG, AZU1, CARD10, CARTPT, CCDC88A, CHRM1, EDN2, GADD45B, GADD45G, GAP43, IRAK1, LYK5, MALT1, MAP3K13, MAP3K4, MAP3K7, MAP3K7IP1, PARD3, PICK1, PPAP2A, PRKD3, TAOK2, TNFSF15, TRAF2, TRAF6, TRAF7, ZAK	0.006
Regulation of angiogenesis	26	AGGF1, AMOT, ANGPTL3, ANGPTL4, BTG1, CHRNA7, COL4A2, COL4A3, FOXO4, HTATIP2, IL17F, NF1, NPPB, NPR1, PF4, PLG, PML, RHOB, RNH1, RUNX1, SERPINF1, SPHK1, SPINK5, STAB1, TNFSF12, TNNI3	0.006
G PROTEIN Signaling coupled to IP3 phospholipase C activating	45	AGTR1, ANG, AVPR1A, AVPR1B, AZU1, C5AR1, CALCA, CCKAR, CCKBR, CHRM1, CHRM2, DRD1, DRD2, EDG2, EDG4, EDG6, EDN2, EDNRA, EDNRB, EGFR, F2RL3, GAP43, GNA15, GNAQ, GRM5, HOMER1, HRH1, HTR2B, IL8RB, LTB4R, MC3R, NMBR, NMUR1, NMUR2, P2RY1, P2RY11, P2RY2, P2RY4, P2RY6, PARD3, PICK1, PLCB2, PPAP2A, PRKD3, TACR1	0.007
Phosphoinositide mediated signaling	48	AGTR1, ANG, AVPR1A, AVPR1B, AZU1, C5AR1, CALCA, CCKAR, CCKBR, CHRM1, CHRM2, DRD1, DRD2, EDG2, EDG4, EDG6, EDN2, EDNRA, EDNRB, EGFR, F2RL3, GAP43, GNA15, GNAQ, GRM5, HOMER1, HRH1, HTR2B, IL8RB, LTB4R, MC3R, NMBR, NMUR1, NMUR2, P2RY1, P2RY11, P2RY2, P2RY4, P2RY6, PARD3, PICK1, PLCB2, PLCE1, PLCH1, PPAP2A, PRKD3, PTAFR, TACR1	0.007
Phospholipase C activation	14	ANG, AVPR1A, AVPR1B, C5AR1, CCKBR, EDG2, EDG4, EDG6, EDNRA, EGFR, GNA15, GNAQ, NMUR1, PLCB2	0.008
Regulation of myeloid cell differentiation	19	ACIN1, ACVR1B, ACVR2A, CALCA, CARTPT, CDK6, ETS1, FOXO3, IL4, INHA, INHBA, LDB1, MAFB, PF4, RUNX1, SCIN, SPI1, ZBTB16, ZNF675	0.009

P value, calculated by using an empirical phenotype-based permutation test procedure, represents the degree to which the pathway is over-represented at the extremes (top or bottom) of the entire ranked list of the total 825 pathways analyzed after comparison DGF versus EGF (for details about the statistical methodology see Subramanian et al. [22]).

In particular 8 pathways resulted up-regulated (*NLS bearing substrate import into nucleus, nuclear transport, nucleocytoplasmic transport, protein import into nucleus, ribonucleotide metabolic process, nuclear import, pyrimidine nucleotide metabolic process, macromolecule localization*), while 7 down-regulated in DGF (*Vasculature development, activation of protein kinase activity, regulation of angiogenesis, G protein signaling coupled to IP3 phospholipase c activating, phosphoinositide mediated signaling, phospholipase c activation, regulation of myeloid cell differentiation*) compared to EGF.

NLS-bearing substrate import into nucleus was the most significantly up-regulated pathway in DGF at T0 (p < 0.001). This pathway included genes encoding for several isoforms of karyopherins, a group of proteins mainly involved in the nucleocytoplasmic transport.

### Identification of DGF-related transcriptomic profiling 24 hours after transplantation

The same statistical strategy used for the microarray analysis at T0 was used to identify pathways differentially expressed in PBMCs isolated at T24 in 7 DGF versus 7 EGF patients.

Bioinformatics revealed that 24 hours after renal transplantation, 5 pathways resulted up-regulated (*NLS bearing substrate import into nucleus, regulation of small GTPase mediated signal transduction, RNA 3 end processing, regulation of ras protein signal transduction and protein import into nucleus*) and 6 down-regulated (*positive regulation of epithelial cell proliferation, rhythmic process, negative regulation of translation, keratinocyte differentiation, negative regulation of cytokine biosynthetic process, negative regulation of biosynthetic process*) in DGF compared to EGF (p < 0.001, False Discovery Rate < 10%) (Table 3).

**Table 3:** Pathways discriminating patients developing delayed graft function (DGF) from those having early graft function (EGF) 24 hours after transplantation (T24)

**Pathway**	**Number of genes**	**Gene symbol**	**p value**
UP-REGULATED IN DGF			
NLS bearing substrate import into nucleus	13	CBLB, FYB, KPNA1, KPNA2, KPNA3, KPNA4, KPNA5, KPNA6, KPNB1, NCKIPSD, RANBP5, RERE, TRPS1	<0.001
Regulation of small GTpase mediated signal transduction	24	ABRA, ALS2, ARF6, ARHGAP27, CDC42BPA, CDC42BPB, CDC42BPG, CENTD2, CENTD3, DMPK, FGD1, FGD2, FGD3, FGD4, FGD5, FGD6, MFN2, NF1, NOTCH2, PLCE1, RAC1, RALBP1, RASGRP4, TSC1	0.005
RNA 3 end processing	10	CPSF1, CPSF3, CSTF1, CSTF2, CSTF3, GRSF1, NCBP1, PABPC1, SLBP, TRNT1	0.006
Regulation of RAS protein signal transduction	19	ABRA, ALS2, ARF6, ARHGAP27, CENTD2, CENTD3, FGD1, FGD2, FGD3, FGD4, FGD5, FGD6, MFN2, NF1, NOTCH2, PLCE1, RAC1, RALBP1, RASGRP4, TSC1	0.009
Protein import into nucleus	48	AKT1, BCL3, BCL6, CBLB, CDH1, CEP57, F2, F2R, FAF1, FLNA, FYB, GLI3, KPNA1, KPNA2, KPNA3, KPNA4, KPNA5, KPNA6, KPNB1, MCM3AP, MDFI, MXI1, NCKIPSD, NF1, NFKBIE, NFKBIL1, NFKBIL2, NLRP12, NLRP3, NOP5/NOP58, NUP205, PDIA3, PPIH, PPP1R10, PTTG1IP, RANBP2, RANBP5, RERE, RPAIN, SMAD3, TGFB1, TNF, TNFSF14, TNPO1, TPR, TRIP6, TRPS1, ZFYVE9	0.009
DOWN-REGULATED IN DGF			
Positive regulation of epithelial cell proliferation	10	EGFR, EPGN, ERBB2, FGF10, LAMA1, LAMB1, LAMC1, NME1, NME2, TGFA	0.003
Rhythmic process	29	AANAT, ARNTL, BMPR1B, CARTPT, CLOCK, CRY1, EGR3, EIF2B2, EIF2B4, EIF2B5, ENOX2, EREG, FOXL2, HEBP1, HTR7, MTNR1A, OPN4, PER1, PER2, PTGDS, SOD1, SPRR2A, SPRR2B, SPRR2C, SPRR2D, SPRR2E, SPRR2F, SPRR2G, TIMELESS	0.004
Negative regulation of translation	23	APBB1, BCL3, EIF2AK1, EIF2AK3, EIF4A3, ELA2, FOXP3, FURIN, GHRL, GHSR, IL10, IL6, INHA, INHBA, INHBB, NDUFA13, NLRP12, PAIP2, PAIP2B, PRG3, SFTPD, SIGIRR, TSC1	0.006
Keratinocyte differentiation	15	ANXA1, CSTA, DSP, EREG, EVPL, IL20, IVL, LOR, NME2, SCEL, SPRR1A, SPRR1B, TGM1, TGM3, TXNIP	0.006
Negative regulation of cytokine biosynthetic process	12	BCL3, ELA2, FOXP3, GHRL, GHSR, IL6, INHA, INHBA, INHBB, NLRP12, SFTPD, SIGIRR	0.008
Negative regulation of biosynthetic process	30	APBB1, BACE2, BCL3, BRCA1, EIF2AK1, EIF2AK3, EIF4A3, ELA2, FOXP3, FURIN, GCK, GHRL, GHSR, GLA, GRM8, IL10, IL6, INHA, INHBA, INHBB, NDUFA13, NLRP12, PAIP2, PAIP2B, PDZD3, PRG3, SFTPD, SIGIRR, SOD1, TSC1	0.009

P value, calculated by using an empirical phenotype-based permutation test procedure, represents the degree to which the pathway is over-represented at the extremes (top or bottom) of the entire ranked list of the total 825 pathways analyzed after comparison DGF versus EGF (for details about the statistical methodology see Subramanian et al. [22]).

Interestingly, also at T24, the top discriminating pathway between DGF *versus* EGF was NLS-bearing substrate import into nucleus (p < 0.001) (Tables 2 and 3).

### Three-dimensional discrimination of DGF from EGF according to NLS-bearing substrate import into nucleus pathway

**Figure 1:**
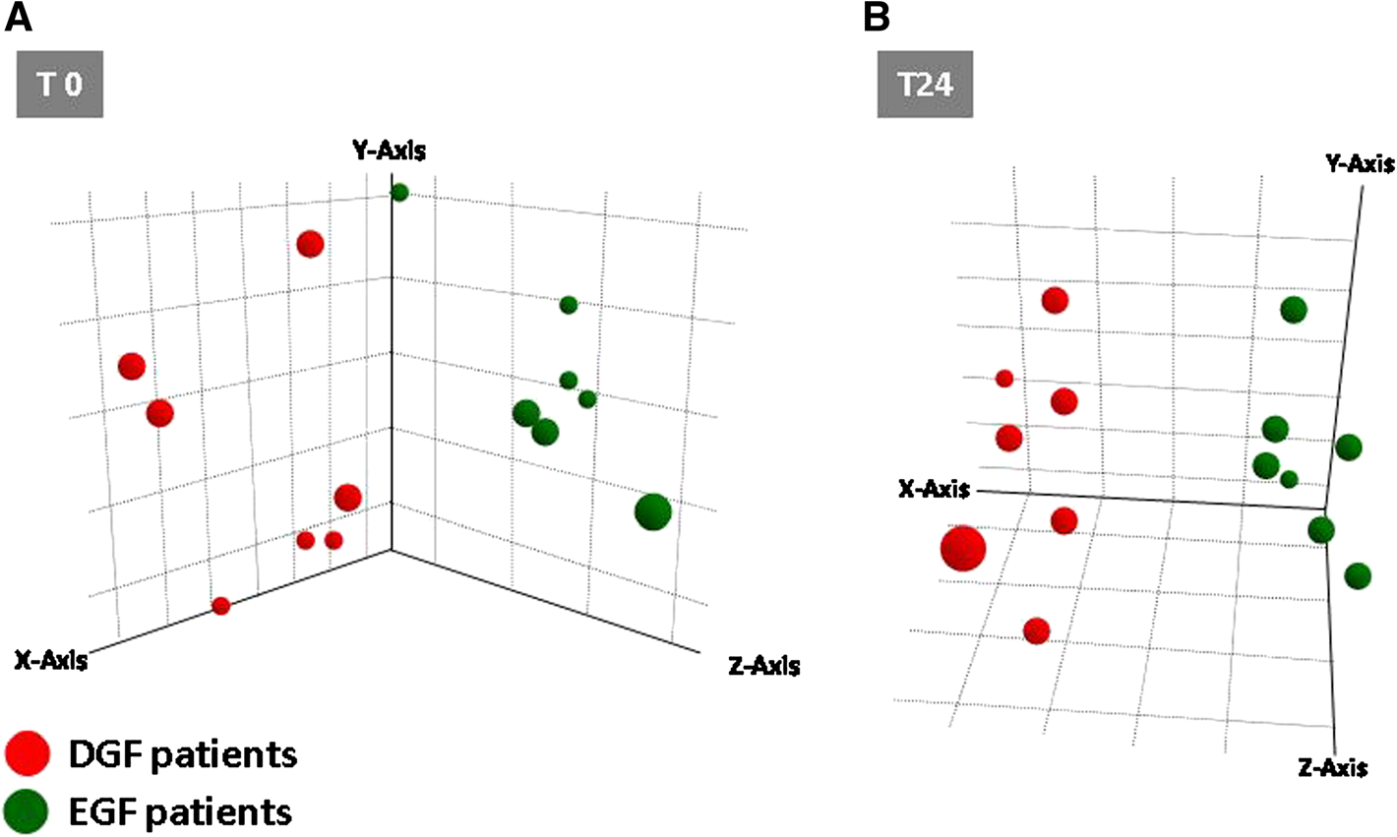
**Principal components analysis (PCA) discriminating renal transplant patients with delayed graft function (DGF) from early graft function (EGF) at the time of transplantation (T0) (A) and 24 hours after transplantation (T24) (B) using the expression level of the genes included in the NLS-bearing substrate import into nucleus pathway.** Both PCA plots were built using the expression level of all 13 genes included in the NLS-bearing substrate import into nucleus pathway. Red dots indicate patients that developed DGF after transplantation and green dots those with EGF. PCA clearly discriminated in three dimensional space the two study groups at both time points.

Principal component analysis using the expression level of all 13 genes included in the *NLS-bearing substrate import into nucleus* pathway was able to clearly discriminate in three dimensional space DGF from EGF at both T0 (Figure 1A) and T24 (Figure 1B).

### Pre-transplant karyopherins-alpha mediated pSTAT3 nuclear migration

To confirm a possible activation of the karyopherin-related machinery in DGF, we analyzed the migration of pSTAT3 from the cytoplasm into the nucleus in PBMCs isolated at T0 from 5 DGF and 5 EGF patients (testing-group) after a short induction (2 and 5 minutes) with IL-6, a well known inducer of this migration process.

The ratio of pSTAT3/STAT3 in PBMCs was significantly higher in in unstimulated cells of DGF compared to EGF patients (more than 10 folds comparing basal levels).

**Figure 2:**
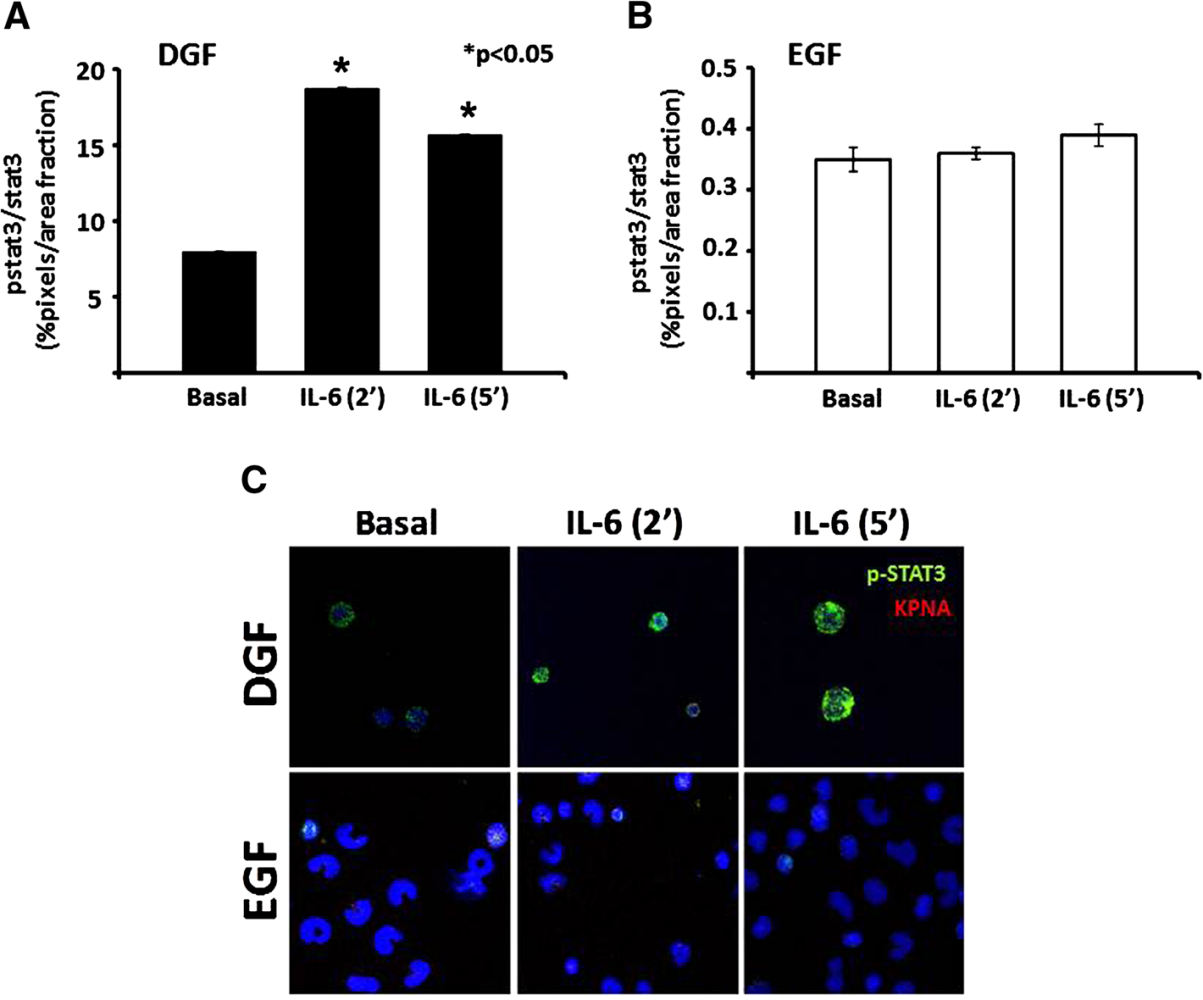
**Phospho-STAT3 (pSTAT3) karyopherins-related nuclear translocation in peripheral blood mononuclear cells (PBMCs) of 5 patients with delayed graft function (DGF) and 5 with early graft function (EGF) after short stimulation with Interleukin (IL)-6. (A and B)** Histograms represent the pSTAT3/STAT3 ratio after 2 and 5 minutes of IL-6 stimulation in PBMCs isolated from 5 DGF and 5 EGF patients, respectively; **(C)** Panels report a representative experiment of the nuclear translocation of p-STAT3 in PBMCs of DGF (upper) and EGF (lower) in basal conditions and after 2 and 5 minutes of IL-6 stimulation. (*) p-value *versus* basal.

Additionally, pSTAT3 translocation and pSTAT3/STAT3 ratio increased rapidly in DGF after short stimulation with IL-6 (Figure 2A and C). On the contrary, in PBMCs isolated from EGF patients, the IL-6 did not induce the aforementioned changes (Figure 2B and C).

Therefore, these findings suggest that the karyopherin-mediated shuttling of intracellular molecules (e.g., transcription factors) may play a role in the onset of DGF in renal transplant patients. However additional studies are needed to confirm and to better address this point.

## Discussion

DGF represents not only the need for continued dialysis, but it is strongly associated with both acute rejection and decreased graft survival [23,24]. In particular, in the long term, patients with DGF are 1.53 times more susceptible to graft loss at 5 years and have an overall 10% lower graft survival rate compared to EGF patients [23-26].

Because of the negative impact of DGF on perioperative care and graft outcome, great efforts have been made to understand the pathogenesis and the biological factors associated with this clinical condition.

Moreover, a variety of clinical algorithms and biological elements have been proposed to predict DGF based on pre-operative risk factors [27-32], but, at the moment, no suitable biomarkers have successfully entered in routine clinical practice.

Additionally, it is unquestionable that, because of a large number of biological factors involved, we are still far from the comprehension of the personal risk factors predisposing to this important clinical complication.

Therefore, it is reasonable that the knowledge of a new piece in the puzzle of DGF could help researchers to identify new potential early diagnostic biomarkers and therapeutic target.

To this purpose, we decided to apply a new analytic microarray strategy to identify biological pathways involved in development of DGF. We chose this new methodological approach because an initial classical “gene by gene” analysis, revealed only weak differences between DGF versus EGF at both T0 and T24. This could be due to the low number of patients included in our study. In fact, other studies utilizing a large dataset, have identified several potential predictive DGF biomarkers with a classical microarray analysis [32,33]. The main property of GSEA is that it finds significant pathways related to the analyzed phenotypic differences by integrating weak association signals that would be lost due to the huge amount of assayed genes. The significance of a pathway was assessed through non-parametric permutation tests that compare the actual normalized enrichment score with the ones obtained by chance. To this end, 1000 random permutations of the phenotypic labels were performed. In the permutation tests the enrichment scores were properly normalized in order to consider the size of the pathways. Moreover, the effects of the multiple comparisons were taken into account by assessing for each pathway the false discovery rate.

However, when we used a customized pathway analysis, we identified 15 pathways (8 up-regulated and 7 down-regulated) and 11 pathways (5 up-regulated and 6 down-regulated) significantly associated with DGF at T0 and T24, respectively. No classical biological elements known to be deregulated in DGF patients have been identified. Only the IL-18, was included in one of the deregulated pathways in DGF at T0 (vasculature development). This result could be due to the fact that our analysis has been performed on RNA extracted from PBMC. In fact, these markers have been primarily studied in urine and tissue samples.

Interestingly, the most significantly up-regulated pathway at both time points was the NLS-bearing substrate import into nucleus. This pathway is involved in the movement of a protein bearing a nuclear localization signal (NLS) from the cytoplasm into the nucleus, across the nuclear membrane.

In detail, this nucleocytoplasmic transport occurs through cylindrical structures spanning the nuclear envelope known as nuclear pore complexes (NPCs) [34]. NPCs are large protein assemblies of approximately 125 MDa in mammalian cells. These structures allow passive exchange of ions, small molecules and small proteins (<20 KDa), but restrict passage of macromolecules to only those bearing appropriate signals. The direction of transport through the NPC is determined by a signal. The NLS directs proteins into the nucleus and the nuclear export signal (NES) directs the transport of proteins toward the cytoplasm.

Karyopherin-alpha (also known as importin-alpha) is an adaptor protein that recognizes the first discovered or classical NLS, which is characterized by one or two stretches of basic amino acid residues [35,36]. Karyopherin-alpha interacts with Karyopherin-beta1 (also known as importin-beta) and together these proteins form a heterodimer which mediates the nuclear import of proteins containing a classical NLS [35]. Our data underlines that our cells show the activation of this machinery.

Previous research showed that members of the signal transducers family and activators of transcription (STAT) use this mechanism for their passage from cytoplasm into the nucleus [37-43]. These molecules, and in particular those involved in the JAK/STAT signaling are primarily involved in the pathogenesis of renal I/R injury [44-47].

Therefore, although there are some limitations (such as small number of patients included in the microarray analysis, absence of the analysis of the predictive power of our identified biological elements and absence of graft histological evaluation) our study shows, for the first time, that karyopherins may have a pivotal role in the development of DGF and these molecules may be new valuable diagnostic predictors. Additionally, they could in future be used as therapeutic targets. In fact, in the last years, several drugs have been studied acting inhibiting karyopherin trafficking (e.g., importazole, Ivermectin) [48-50].

## Conclusions

In conclusion, the present study, utilizing an innovative transcriptomic approach, suggests the involvement, immediately before transplantation, of karyopherin-mediated nuclear transport in the onset and development of DGF. Additionally, although further studies are needed, it reveals that the analysis of this condition could represent a new potential clinical tool useful as predictive and early diagnostic DGF biomarker in renal transplantation.

## Competing interests

The authors declare that there is no conflict of interests regarding the publication of this article.

## Authors’ contributions

GZ, FR and GG designed the research; GZ, FR, PP, SG, PS and MA performed the experiments; GZ, FR, GG, SG, NA, LG, AL wrote the manuscript; GZ had primary responsibility for final content. All authors read and approved the final manuscript.

## Pre-publication history

The pre-publication history for this paper can be accessed here:

http://www.biomedcentral.com/1755-8794/7/14/prepub
